# Early-Onset Chondrocalcinosis With Erosive Progression Following Trauma: A Case Report

**DOI:** 10.7759/cureus.75469

**Published:** 2024-12-10

**Authors:** Angelo Nigro

**Affiliations:** 1 Rheumatology Department of Lucania, "Madonna delle Grazie" Hospital, Matera, ITA

**Keywords:** calcium pyrophosphate crystal deposition, chondrocalcinosis, early onset, erosive progression, joint trauma, pseudogout

## Abstract

Chondrocalcinosis, commonly associated with aging, is characterized by the deposition of calcium pyrophosphate dihydrate (CPPD) crystals in cartilage and periarticular tissues. Early-onset cases are rare and not well-documented. We report a case of a 60-year-old woman with a probable onset of CPP deposition (CPPD) disease during adolescence, presenting with inflammatory flare-ups and erosive progression following minor trauma. This case highlights the atypical presentation of chondrocalcinosis in a younger individual and emphasizes the potential for erosive joint damage, contributing to the understanding of disease progression and management strategies in similar patients.

## Introduction

Chondrocalcinosis, also known as pseudogout, is a rheumatologic condition characterized by the deposition of CPP (CPPD) in articular cartilage, leading to joint inflammation and degeneration [1]. It typically affects the elderly population, with incidence increasing significantly after the age of 60 [2]. Early-onset chondrocalcinosis is uncommon and often associated with metabolic disorders or genetic predispositions [3]. Erosive changes in chondrocalcinosis are rare and not as extensively studied as in other arthropathies like rheumatoid arthritis [4]. We present a unique case of early-onset chondrocalcinosis with erosive progression triggered by trauma, aiming to shed light on its clinical course and implications for management.

## Case presentation

A 60-year-old woman presented to our rheumatology clinic with persistent pain and limited mobility in her left wrist. She reported a history of intense arthralgia in her left hand at the age of 16, which led to hospitalization, although no swelling was noted at that time. Unfortunately, she did not retain any medical records from that period.

Over the subsequent years, she experienced progressive osteoarthritic changes in several distal interphalangeal (DIP) joints of both hands, accompanied by persistent pain and reduced range of motion in the left wrist. One year before she visited our clinic, she noticed a worsening of symptoms following a minor wrist trauma.

**Figure 1:**
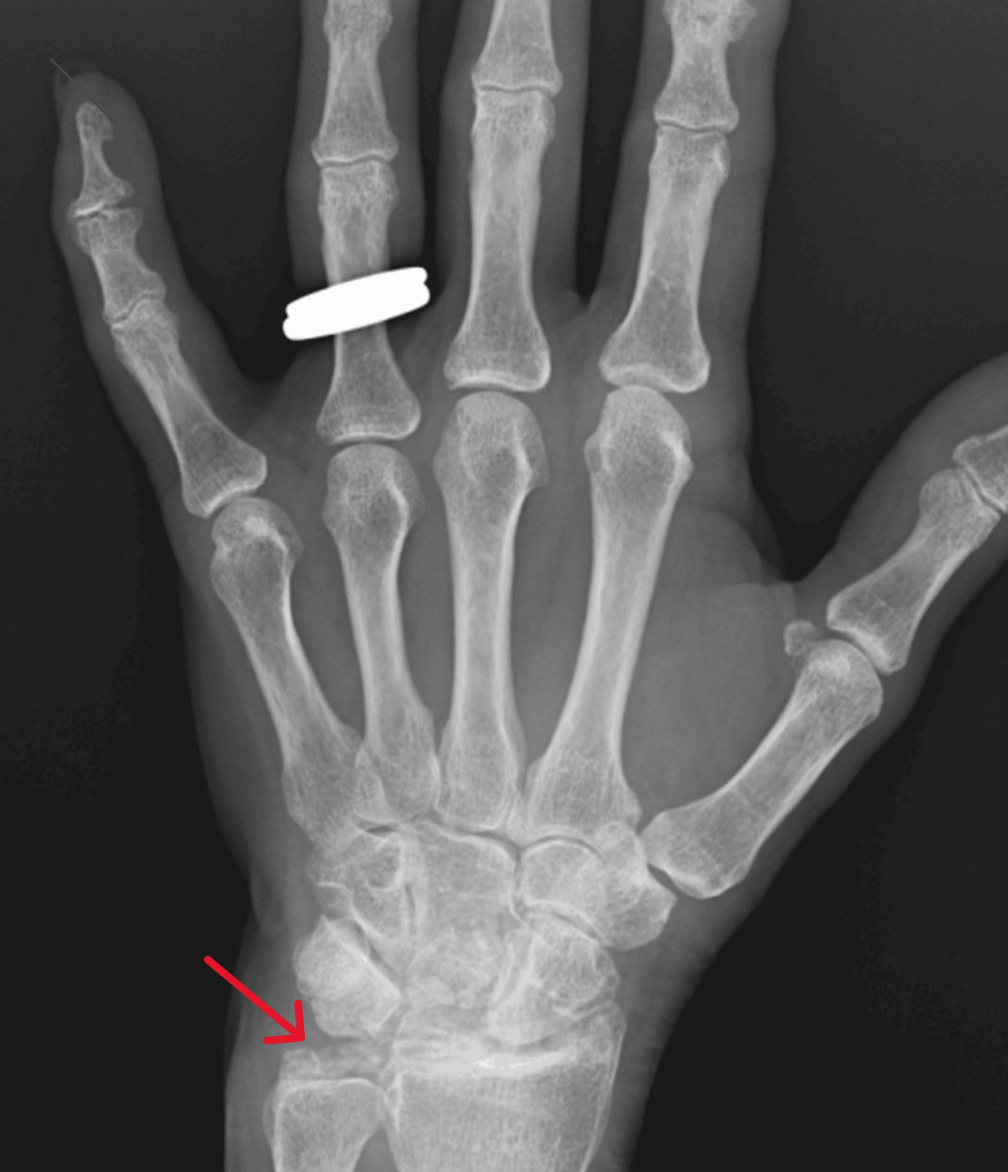
X-ray of the left hand and wrist from 2023, showing the presence of arthritic changes and evident calcifications of the triangular ligament on the left

On examination, there was mild swelling and marked limitation of movement in the left wrist. She provided a radiograph taken a year earlier, which revealed severe osteoarthritic changes in the left wrist, significant narrowing of joint spaces, calcification of the triangular fibrocartilage complex, and diffuse cystic and erosive lesions (Figure 1).

Laboratory investigations, including inflammatory markers (erythrocyte sedimentation rate and C-reactive protein), antinuclear antibodies (ANA), and anti-cyclic citrullinated peptide antibodies, were within normal limits or negative. There was no evidence of inflammatory involvement in other joints.

**Figure 2:**
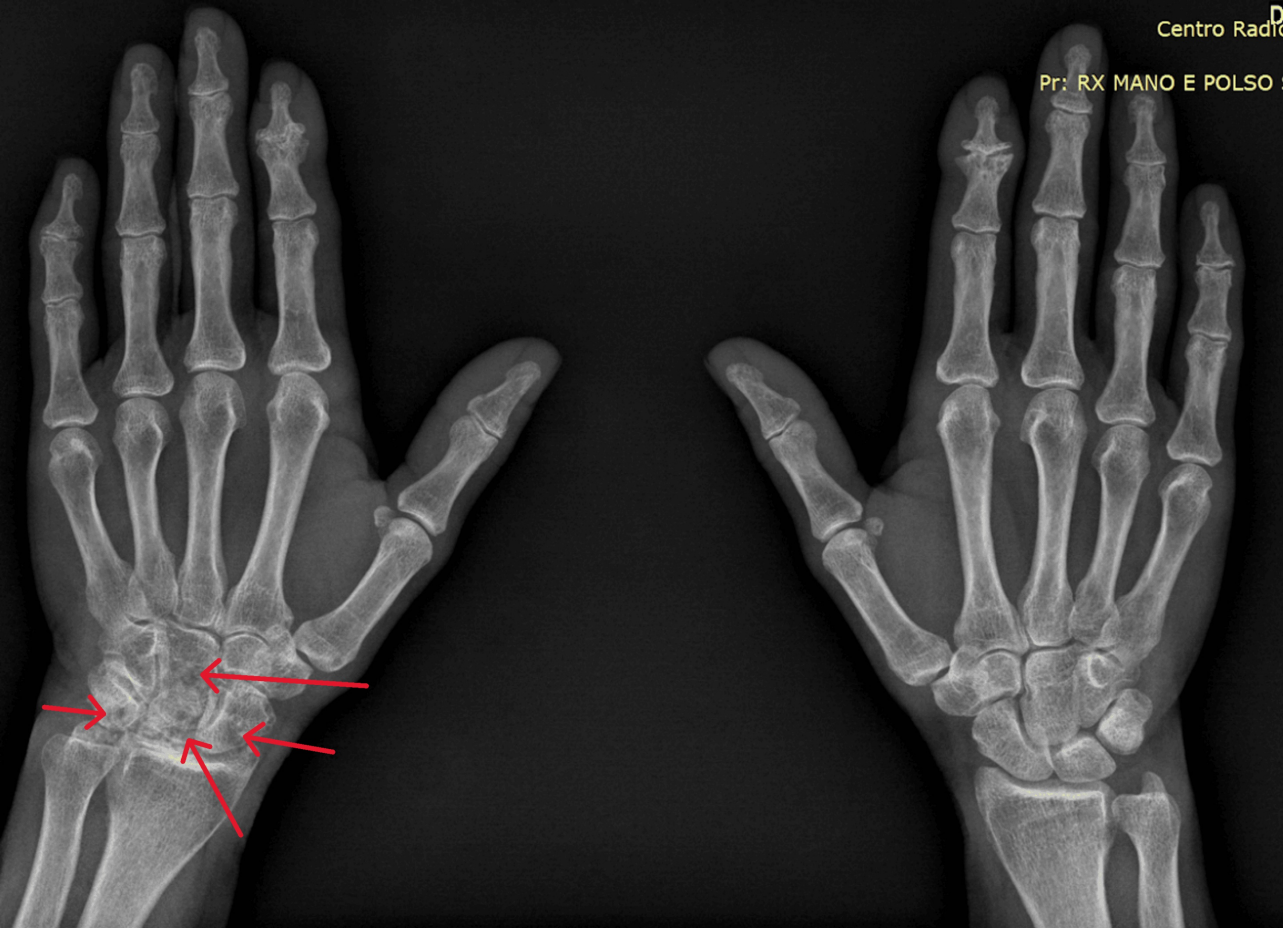
Follow-up X-ray of the left hand after one year, showing progression of radiographic damage in the left wrist with new erosions and geode formations

**Figure 3:**
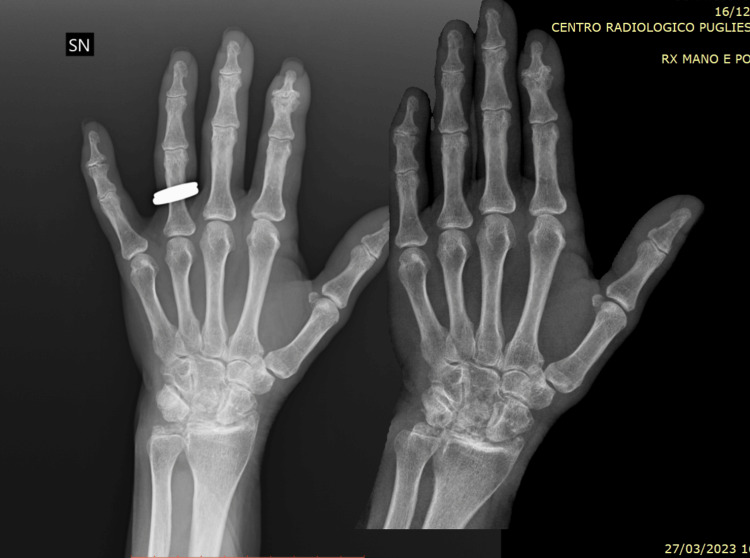
Comparison of two radiographs of the left hand and wrist taken one year apart, demonstrating visible progression of radiographic damage at the wrist, with the development of new erosions and geodes

The patient was commenced on colchicine and low-dose 6-methylprednisolone, which provided symptomatic relief. A follow-up radiograph was recommended, which demonstrated the progression of joint damage with new erosive lesions compared to the previous imaging (Figures 2, 3).

## Discussion

This case illustrates an unusual presentation of chondrocalcinosis with probable onset during adolescence and an erosive course exacerbated by trauma. Early-onset chondrocalcinosis is rare and often linked to metabolic disorders such as hemochromatosis, hyperparathyroidism, hypomagnesemia, or familial CPP deposition disease [3]. However, our patient had no clinical or laboratory evidence of these conditions.

The erosive nature of the disease in this patient is noteworthy. While CPP deposition can lead to joint degeneration, significant erosive changes are uncommon [4]. Trauma has been suggested as a precipitating factor for acute pseudogout attacks [5], and in this case, may have contributed to both symptomatic exacerbation and accelerated joint damage.

The absence of systemic inflammation markers and negative serology for rheumatoid arthritis and other autoimmune conditions helped in narrowing the differential diagnosis. Imaging played a crucial role in identifying the characteristic calcifications and erosive changes associated with chondrocalcinosis [6].

Management of chondrocalcinosis focuses on symptom relief and preventing joint damage. Colchicine and corticosteroids are effective in controlling acute flare-ups [7]. However, the progressive nature of the disease in this patient despite therapy highlights the need for close monitoring and possibly more aggressive interventions.

## Conclusions

This case emphasizes the importance of considering CPPD in younger patients presenting with joint pain and radiographic evidence of calcifications, even in the absence of typical risk factors such as advanced age, metabolic disorders, or previous joint disease. Recognizing CPPD in this population is crucial, as misdiagnosis or delayed diagnosis can lead to the progression of the disease and potentially irreversible joint damage. The potential for erosive joint damage, particularly following trauma, highlights the need for a high index of suspicion among clinicians and the use of advanced imaging techniques when standard radiographs are inconclusive. Early diagnosis and appropriate management, including possible lifestyle adjustments, physical therapy, and pharmacologic intervention, may prevent significant morbidity, improve the quality of life for affected individuals, and reduce the long-term burden on healthcare systems.
